# Work Ability after Breast Cancer: Study of Healthcare Personnel Operating in a Hospital of South Italy

**DOI:** 10.3390/ijerph191710835

**Published:** 2022-08-31

**Authors:** Francesca Vella, Veronica Filetti, Luigi Cirrincione, Venerando Rapisarda, Serena Matera, Alenka Skerjanc, Emanuele Cannizzaro, Ermanno Vitale

**Affiliations:** 1 Occupational Medicine Section, Department of Clinical and Experimental Medicine, University of Catania, 95124 Catania, Italy; 2 Department of Sciences for Health Promotion and Mother and Child Care “Giuseppe D’Alessandro”, University of Palermo, 90127 Palermo, Italy; 3 Clinical Institute for Occupational, Traffic and Sports Medicine, University Medical Centre Ljubljana, 1000 Ljubljana, Slovenia

**Keywords:** breast cancer, HCWs, occupational medicine, work ability

## Abstract

Breast cancer (BrC) treatment can produce disabilities that often impact the quality of daily life and impact the social and working relationships of the patient. This paper looked into the remaining work ability in a group of female healthcare personnel (HCPs) with BrC in Southern Italy. Each HCP was subjected to a medical check, routine blood tests, and a questionnaire on the work ability index (WAI). Of 980 (100%) HCWs undergoing health control, 6% (*n* = 54) had experienced BRC, and only 66.6% (*n* = 36) agreed to take part in the study. A total of 28 (78%) were on night shifts. The WAI score was quite low in 5 (13.8%) cases, moderate in 10 (27.7%) cases, good in 14 (38.8%) cases, and excellent in 7 (19.5%) HCWs. Among all health figures, in nurses as well as technical staff, lower WAI scores were observed. HCWs reported various comorbidities, which affected WAI score, such as limited mobility in the upper limbs, arm/shoulder pain, numbness, and lymphoedema. The main complication that negatively affects any work activity is the morbidity in the upper limbs. This seems to affect the ability to perform tasks, and the re-entry to work is highlighted on sick leave days.

## 1. Introduction

Breast cancer (BrC) is one of the most widespread health issues worldwide [1,2], covering 30% of cancers in women in Italy [2,3,4,5].

It is also known as the second cause of death in developed countries and the third main cause of death in less developed countries [4]. In 2019, about 53,000 new cases of female BrC were diagnosed in Italy [5]. According to the World Health Organization (WHO) prediction, up to 2.3 million women will be diagnosed with BrC by 2050 [2,6]. BrC represented the first cause of death in the different age spans of life, accounting for 28% before the age of 50 years, 21% ranging between 50 and 69 years, and 14% after the age of 70 years [5]. Today’s, average survival, 5 years after the diagnosis, is 87%, while 10 years after the diagnosis, the average survival is 80% in all cases [5]. 

Early BrC is considered potentially treatable. Therapy progressed remarkably in the last few years, with less ICUs occupied, both for locoregional and systemic therapy; avoiding overtreatment, but also undertreatment, is often preferred as a protocol choice [7]. BrC is very often disabling from the working point of view, in particular, it may impair patients ability to meet the physical and mental demands of a job. Return to work after cancer treatment is an important event for the patients. It is a considerable sign of recovery for patients and is often experienced as a transition from considering oneself a patient to functioning normally again [7]. 

Several factors affect the BrC incidence: lifestyle; increased life expectancy; changes in reproductive patterns; increasing obesity; the socio-cultural impact; and improved early diagnosis, mainly due to efficient screening protocols [5,6,7,8,9]. 

Several occupational risk factors are recognized for BrC, and these are often present in healthcare facilities: ionizing radiation, ethylene oxide, and shift/night/work [9,10,11,12,13,14,15,16,17,18].

According to the scientific population, correlations exist between night shift work and an increased incidence of BrC [19,20]. Indeed, night work is classified by the International Agency for Research on Cancer (IARC) as a Group 2A probable human carcinogen [9,11,16], in that it is capable of disrupting the normal heart rhythm [21,22,23,24].

BrC therapy frequently means surgical treatment combined with chemo- and radio-therapy [25]. These last are particularly destructive, as they often cause localized and systemic impairment, which also cause severe disabilities [25]. Infirmities resulting from BrC therapy frequently worsen patients’ quality of life and impact both social and working life [26,27,28,29,30].

Several surveys already pinpointed that BrC women patients in lower working classes need longer resting periods from work and show a greater loss of working skills [31,32].

The present study explored the remaining work ability in a set of female healthcare personnel (HCPs) with BrC in Southern Italy.

## 2. Materials and Methods

This retrospective case control study was carried out in 2019–2020 and involved previously (in the last 20 years) BrC-diagnosed female HCPs operating at a hospital in Southern Italy.

Exclusion criteria were: being affected by other systemic pathologies, such as cardiovascular, metabolic disease, and not being retired. HCPs invited to participate in the present study were informed about aims and procedures. Subjects voluntarily adhered to the study by signing an informed consent. The study was directed in line with Helsinki Declaration guidelines and while the occupational physician was performing his/her duty according to the Italian law decree 81/2008 [33,34].

For each participant, family, medical, and work histories were carefully carried out to highlight any work-related exposure that potentially fostered the onset of cancer. We investigated demographic factors and lifestyle-related risk elements of each participant, and the activities they participated in during their spare time were also taken into account.

Furthermore, the oncology agenda was asked of each participant, updated to the latest treatment, as well as the latest checks performed, such as the genetic, histological, and diagnostic imaging tests [4,35]. During the medical examination, parameters such as body mass index (BMI), smoking habit, alcohol habit (almost 1 glass of alcoholic beverage per day), workplace, parity, family history of BrC, hormonal therapy (progestin or estroprogrestinic contraceptive therapy), and breastfeeding were detected.

Each participant was subjected to medical check, routine lab tests, and a questionnaire on their work ability index (WAI) [36]. 

The WAI is an index used to evaluate an operator’s individual work ability so as to establish working skills according to age, pathologies, etc. [37]. The calculation of the WAI scores was founded on the Finnish Institute of Occupational Health (FIOH) protocol [37,38]. The WAI answers 7 questions looking into the extents below: (0–10 points) present working capacity compared with one’s best life period; (2–10 points) ability to work concerning the job requirements; (1–7 points) diagnosed pathologies; (1–6 points) reduction in working capacity due to illness, estimated by the individual; (1–6 points) sick leave over the past 12 months; (1–7 points) personal expectations of one’s work skills two years onwards; (1–4 points) psychological conditions/resources [39,40,41]. WAI score (7–49) may be: low (7–27 score); moderate (28–36 score); good (37–43 score); or excellent (44–49 score) [36,37,40].

A control group of HCPs, in the same hospital, was arranged for anthropometrical characteristics, job career, workplace, and habits. The control group consisted of women without previous BrC diagnosis and still in service. The exclusion criteria were the same as the observational group. 

### Statistical Analysis

The collected data were included in a bespoke database. Descriptive statistics were carried out to describe the sample of the study. Data were tested for normality with the Kolmogorov–Smirnov test. All variables were not normally distributed. The association between the different variables was analyzed with the chi-square test (X_2_) or Fisher’s exact test, and Student’s *t*-test. The suitable relationship measures were assessed through the odds ratio (OR; 95% CI). The significant variables of the univariate analysis and logistic regression models were used. The variables were considered statistically significant when *p* < 0.05. Statistical study was carried out with SPSS software (IBM SPSS Statistics for Windows, Version 23.0. Armonk, NY, USA: IBM Corp).

## 3. Results

Out of the 980 (100%), HCPs being health checked in 2019–2020, 54 (5.5%) suffered from BrC (inclusion criteria). BrC identifies were set in the 2002–2019 period. 

**Figure 1:**
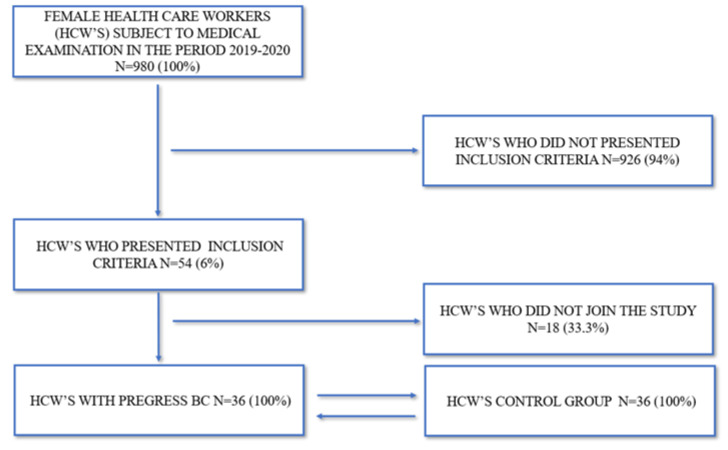
Flow chart of the sample studied.

A total of 36 of these 54 HCPs (66.6%) agreed to take part in the study. Out of the 18 HCWs who did not belong in the study, 11 (61%) refused for work for reasons that are due to lack of time, and 7 (39%) did not want to retrace a dramatic episode in their lives (see Figure 1).

Participants were, on average, 52.9 ± 7.3 years old, with a service duration of 26.2 ± 7.4 yrs. The BMI average was 25.3 ± 4.8, equivalent to obesity. Most of the participants were nurses in both groups. Five HCPs (13.5%) were still in the fertile period; among controls there were four (11.3%) in the fertile period. The average number of children was 2.04 ± 0.55 in cases and 2.22 ± 1.31 in the control group. The sample characteristics are described in Table 1.

There resulted a statistically significant difference between the cases and the controls in the family history for BrC and the hormone therapy. When diagnosed, the HPCs’ mean age was about 45 years, and the mean service career was 17 years. Most cases were shift workers, including night workers, and most of them used oral birth control devices.

The workplace risk assessment identified the following health risks for cases: 36 (100%) biological risk; 13 (36%) video display units; 21 (58%) hand lifting of patients/load; and 28 (78%) night shift work. Anyone was exposed to chemical carcinogens, by their knowledge.

Studying the risks, only a significant correlation with night shift work was found: OR = 1.51, CI 95% (1.47–1.56).

The investigation of health records concerning BrC exhibited that the majority of cases had BrC histologically classified as Luminal-A (ER+ and/or PgR +HER2−).

Luminal-A type, quadrantectomy, lymphadenectomy, chemotherapy, and radiotherapy prevail in cases of BrC. Table 2 reports the therapy adopted concerning the histological classification.

According to the tumor–nodes–metastasis (TNM) classification [42], stages I and IIA were luckily the most frequent. Table 3 shows the subdivision of the sample according to the TNM classification.

Only 14 (38.8%) cases had a follow-up with periodic checks every 6 months; 19 HCWs (53%) had BrC over 5 years before and were currently under screening protocols. When the questionnaire was given, one case was under chemotherapy again, with distant lymph node and bone metastases.

All 36 (100%) cases had gone back to work. The mean number of days of absence from work was 155.8 ± 205.4 days for nurses/technicians, compared to 128.2 ± 239.7 days for doctors/biologists. Comparing the return to work of nurses/technicians with medical/biologists, the first required an average longer time, but not statistically significant, compared to the latter.

WAI average score was good in cases with BrC (37.16 ± 7.6), but lower than those with the control group (38.2 ± 7.7). Table 4 reports the results of the WAI questionnaire to the treatment chosen. The figures observed in BrC subjects were significantly lower than those found in the control group. Therefore, HCPs group with previous BrC, had had a good functional recovery. 

From the data analysis, it is observed that HCPs who underwent mastectomy (*n* = 10) had a lower WAI score than those who had a quadrantectomy (*n* = 26); moreover, only 4 (12%) HCPs were treated with quadrantectomy and underwent radiotherapy. The results of the WAI score of the last group were greater than 50% of the total values. Furthermore, the subjects undergoing quadrantectomy + chemo/radiotherapy had a higher WAI score than those who had the mastectomy + chemo/radiotherapy. HCPs who underwent lymphadenectomy in addition to the main surgical treatment showed WAI scores less than 50% of the values.

The nurses and technical staff revealed lower WAI scores than other health workers. Limited mobility in the upper limbs, arm/shoulder pain, numbness, and lymphoedema were the main comorbidities denounced by the HCPs that affected WAI score.

## 4. Discussion

The purpose of this survey was to analyze a group of HCPs with prior BrC, to assess residual working skills concerning age and pathology, considering the return to work as an important part of the recovery process. 

The mean age of the diagnosed sample was around 46 years, following the scientific literature data, which identifies the post-menopausal age as the most at risk [31]. 

From the analysis of the sample, 78% of the cases were shift workers. Lifestyle-related risk habits, such as alcohol and smoking, were not very significant, probably because of the low occurrence in the sample.

As in the literature, the OR analysis confirms that night work seems to be a BrC risk factor [43,44,45], according to the IARC, which claims that night shift work is likely to be carcinogenic to humans (Group 2 A) [16].

Nurses and technicians revealed a higher frequency of BrC than other healthcare operators. They remained absent from work for longer periods than doctors/biologists. As it turns out, the professional risks nurses run are different, that is, they carry out more physically demanding jobs, such as manual handling of loads, lifting, replacing, etc. [31,32]. The results of WAI questionnaire analysis confirm higher scores in doctors and biologists than nurses and technicians.

A significant variation was also seen in the WAI index score regarding the type of treatment: the most incapacitating one (mastectomy + chemotherapy + radiotherapy) led to a huge presence of side effects that affected the working ability. The type of treatment received, surgery, radiotherapy, chemotherapy, and hormone therapy, significantly affected recovery times and their going back to work; according to Gregorowitsch et al., (2019), patients reported a severely reduced working capacity during BrC treatment and further reduced when undergoing chemotherapy or lymphadenectomy [7,46]. It turned out that chemotherapy was the most incapacitating treatment, prolonging the sick leave because of its side effects. 

Limited mobility in the upper limbs, arm/shoulder pain, numbness, and lymphoedema are the main comorbidities detectable after therapy [47,48]. They seem to impact the return to work, which is evident in the sick leave days and in the ability to perform one’s job [49]. Only a few studies analyzed residual working skills upon returning to work [49]. Postsurgical pain, rotator cuff disease, adhesive capsulitis, arthralgias, cervical radiculopathy, brachial plexopathy, mononeuropathy, postmastectomy pain syndrome, lymphedema, axillary web syndrome, deep vein thrombosis, and cellulitis commonly cause upper body pain and dysfunction in BrC. Diagnostic specificity is a key first step to safely and effectively restoring the function and quality of life of BrC survivors [50].

Limited mobility in the upper limbs, arm/shoulder pain, numbness, and lymphoedema are the main comorbidities reported by the HCPs (*n* = 16) undergoing lymphadenectomy, with a negative impact on everyday life activities, as well the psychological sphere and their working activities. Morbidity in the upper limbs is one of the main complications that can negatively impact working activity, the psychosocial sphere, and generally the quality of life [44,50] of workers.

Since our participants fell in the economically active domain, an early return, apart from helping the recovery of the worker’s health conditions, would further aggravate her workability.

About half of our cases had a stage of cancer 0–1 at diagnosis time. We may deduce this is the result of important screening protocols that, over the years, allowed us to realize a diagnosis of the pathology at an increasingly earlier stage. 

We know that the work ability of cancer patients is severely impaired in the first months after the first day of sick leave, but it significantly improves in the following months. Self-assessed work ability after 6 months proved to be a strong predictor of later return to work in cancer survivors independently of age and therapy [51].

Scientific data suggest that a multidisciplinary approach is to be preferred. Occupational health doctors should take note of individual and collective risk assessments, promote a healthy lifestyle before and after sick leave, as well propose rehabilitation and solutions to improve the interactions between employees and the work milieu [17,52,53]. 

Encouraging patients with low-stage diagnoses to return to work would be advisable, enhancing their quality of life and reducing days of sick leave and requests for disability pension. High-stage patients might take more time to recover from chemotherapy; however, they should be encouraged if their general condition allows for it [17,26,54]. 

The strengths of the study were having made a contribution regarding the type of treatment and WAI. The study costs were very low, so the study can be reproduced on any operational context without spending problems.

The weaknesses of the research were the sample size, as it was small and consisted mainly of well-educated people. Only a questionnaire was used to assess work ability. Only Caucasian women, predominately well-educated, were present in the sample. However, the results provide initial evidence of a relationship between BrC and shift work. Future studies should include larger and more diverse samples.

## 5. Conclusions

In conclusion, the type of treatment received, surgery, radiotherapy, chemotherapy, and hormone therapy, significantly affected the recovery times of patients and their going back to work. The relationship between shift work, including night work, and BrC inception is confirmed also by this study. Additionally, it is important to return to work as soon as possible, as for the worker it means a return to normal life and social reintegration. Occupational physicians can improve their key role at the workplace and in society [26]. Early diagnosis results in less disturbing procedures and also in the possibility of ruling out highly incapacitating treatments, such as chemotherapy [25,42]. This is emphasized in our study by significantly higher WAI scores when less aggressive treatment is administered. Higher residual working abilities are making an earlier return of BrC female patients to their work environment possible. 

## Figures and Tables

**Table 1:** Sample main characteristics.

	Cases with Prior BrC 36 (100%)	Control Group 36 (100%)	*p*-Value
Average age (yrs)	52.9 ± 7.29	53.7 ± 7	n.s.
Age (at the time of diagnosis)	45.2 ± 7.2	-	n.c.
Menarche (age)	11.8 ± 1.09	11.7 ± 1.2	n.s.
Menopause (age)	44.7 ± 4.9	44.2 ±4.7	n.s.
Service duration (yrs)	26.1 ± 7.5	25.9 ± 6.7	n.s.
Job time (yrs–at diagnosis)	17.7 ± 8.8	15.7 ± 7.1	n.s.
Shift workers	28 (78%)	20 (55.6%)	n.s.
BMI mean (kg/m^2^)	25.3 ± 4.8	25.1 ± 2.7	n.s.
BMI < 18.5	5 (14%)	7 (19%	n.s.
BMI 18.5–24.9	12 (33%)	6 (17%)	n.s.
BMI 25–29.9	15 (42%)	17 (47%)	n.s.
BMI > 30	4 (11%)	6 (17%)	n.s
Smokers	7 (19.4%)	5 (13.8%)	n.s.
Cigarette packages/year	14.5 ± 2.5	15.1 ± 2.1	n.s.
Daily alcohol intake (1 glass per day)	2 (5.5%)	3 (8.3%)	n.s.
Medical Doctors/biologists	15 (41.6%)	14 (38.8%)	n.s.
Nurses/technicians	21 (58.4%)	22 (61.1%)	n.s.
Surgical area	10 (28%)	9 (25%)	n.s.
Medical area	14 (39%)	16 (44.4%)	n.s.
Service area	12 (33.3%)	11 (30.6%)	n.s.
Nulliparous	11(30.5%)	9 (25%)	n.s.
BrC family history	11 (30.5%)	2 (5.5%)	*p* < 0.05
Hormonal contraception	13 (36.1%)	0	*p* < 0.05
Breastfeeding	22/25 (61.1%)	18/27 (50%)	n.s.

n.s. not significant, n.c. not calculable.

**Table 2:** Therapy performed in relation to the cancer histological classification.

	Luminal-A	Luminal-B	Her 2+
N° cases	24 (67%)	4 (11%)	8 (22%)
MCT	5 (21%)	0	5 (62.5%)
QDCT	19 (79%)	4 (100%)	3 (37.5%)
LMCT	12 (50%)	4 (100%)	6 (75%)
CTP	/	/	/
RDT	4 (16%)	/	/
CTP and RDT	20 (84%)	4 (100%)	8 (100%)

Quadrantectomy = QDCT; chemotherapy = CTP; radiotherapy = RDT; lymphadenectomy = LMCT; and mastectomy = MCT.

**Table 3:** TNM staging and classification of the case with BrC.

**STAGE 0**	Tis	N0	M0	4 (11%)
**STAGE I**	T1	N0	M0	11 (30.5%)
**STAGE II A**	T0	N1	M0	/
	T1	N1	M0	8 (22%)
	T2	N0	M0	2 (5%)
**STAGE IIB**	T2	N1	M0	4 (11%)
	T3	N0	M0	3 (8.3%)
**STAGE III A**	T0	N2	M0	/
	T1	N2	M0	2 (5%)
	T2	N2	M0	1 (2.7%)
	T3	N1, N2	M0	1 (2.7%)
**STAGE III B**	T4	N1, N2, N3	M0	/
**STAGE III C**	Any T	N3	M0	/
**STAGE IV**	Any T	Any N	M1	1 (2.7%)

**Table 4:** Results of the WAI questionnaire in relation to the treatment adopted on cases with BrC.

WAI Score	7–27 (Low)	28–36 (Moderate)	37–43 (Good)	44–49 (Excellent)
QDCT + CTP/RDT	-	2 (6%)	3 (8%)	4 (11%)
QDCT + RDT	-		2 (6%)	2 (6%)
QDCT + CTP	-	-	-	-
QDCT + CTP/RDT + LMCT	4 (11%)	5 (14%)	4 (11%)	-
QDCT + RDT + LMCT	-	-	-	-
MCT + CTP	-	-	-	-
MCT + CTP/RDT + LMCT	3 (8%)	6(16%)	-	-
MCT + LMTC	-	-	-	-
MCT + CTP/RDT	1 (3%)	-	-	-
MCT + RDT	-	-	-	-
MCT + LMCT + RDT	-	-	-	-
TOTAL STUDY GROUP	8 (22%) *	13 (33%) *	9 (23%) *	8 (22%) *
CONTROL GROUP	2 (6%) *	6 (16%) *	20 (51%) *	9 (23%) *

* Significantly for *p* < 0.5; quadrantectomy = QDCT; chemotherapy = CTP; radiotherapy = RDT; lymphadenectomy = LMCT; and mastectomy = MCT.
